# Development of a prediction model for cognitive impairment of sarcopenia using multimodal neuroimaging in non‐demented older adults

**DOI:** 10.1002/alz.14054

**Published:** 2024-06-18

**Authors:** Sunghwan Kim, Sheng‐Min Wang, Dong Woo Kang, Yoo Hyun Um, Han Min Yoon, Soyoung Lee, Yeong Sim Choe, Regina EY Kim, Donghyeon Kim, Chang Uk Lee, Hyun Kook Lim

**Affiliations:** ^1^ Department of Psychiatry Yeouido St. Mary's Hospital, College of Medicine, The Catholic University of Korea Seoul Republic of Korea; ^2^ Department of Psychiatry Seoul St. Mary's Hospital, College of Medicine, The Catholic University of Korea Seoul Republic of Korea; ^3^ Department of Psychiatry St. Vincent's Hospital, College of Medicine, The Catholic University of Korea Seoul Republic of Korea; ^4^ Department of Rehabilitation Yeouido St. Mary's Hospital, College of Medicine, The Catholic University of Korea Seoul Republic of Korea; ^5^ Department of Psychiatry Brigham and Women's Hospital Boston Massachusetts USA; ^6^ Department of Psychiatry Harvard Medical School Boston Massachusetts USA; ^7^ Research Institute Neurophet Inc. Seoul Republic of Korea; ^8^ CMC Institute for Basic Medical Science the Catholic Medical Center of The Catholic University of Korea Seoul Republic of Korea

**Keywords:** amyloid retention, cognitive impairment, cortical thickness, path model analysis, sarcopenia, white matter hyperintensity

## Abstract

**INTRODUCTION:**

Despite prior research on the association between sarcopenia and cognitive impairment in the elderly, a comprehensive model that integrates various brain pathologies is still lacking.

**METHODS:**

We used data from 528 non‐demented older adults with or without sarcopenia in the Catholic Aging Brain Imaging (CABI) database, containing magnetic resonance imaging scans, positron emission tomography scans, and clinical data. We also measured three key components of sarcopenia: skeletal muscle index (SMI), hand grip strength (HGS), and the five times sit‐to‐stand test (5STS).

**RESULTS:**

All components of sarcopenia were significantly correlated with global cognitive function, but cortical thickness and amyloid‐beta (Aβ) retention had distinctive relationships with each measure. In the path model, brain atrophy resulting in cognitive impairment was mediated by Aβ retention for SMI and periventricular white matter hyperintensity for HGS, but directly affected by the 5STS.

**DISCUSSION:**

Treatments targeting each sub‐domain of sarcopenia should be considered to prevent cognitive decline.

**Highlights:**

- We identified distinct impacts of three sarcopenia measures on brain structure and Aβ.
- Muscle mass is mainly associated with Aβ and has an influence on the brain atrophy.
- Muscle strength linked with periventricular WMH and brain atrophy.
- Muscle function associated with cortical thinning in specific brain regions.
- Interventions on sarcopenia may be important to ease cognitive decline in the elderly.

## 1 BACKGROUND

Sarcopenia refers to the age‐related loss of muscle mass, strength, and function associated with aging, which leads to reduction in the quality of life and increased risk of mortality in the elderly.^1^ Although sarcopenia is primarily associated with physical consequences such as decreased mobility and increased risk of falls, evidence also suggests a connection between sarcopenia and cognitive impairment.^2^ The prevalence of cognitive impairment in patients with sarcopenia was estimated to range from 9.9% to 40.4% in individuals aged ≥ 60 years.^3^ In a previous meta‐analysis, older adults with a diagnosis of sarcopenia had a two‐fold greater risk of cognitive decline.^4^ In addition, cognitive impairment domains, such as processing speed and executive function, have been linked to sarcopenia‐related slow gait speed in community‐dwelling older adults.^5^ While there is evidence to suggest a link between sarcopenia and cognitive impairment, the underlying pathophysiological mechanisms in this relationship are not yet clear.

To date, myokines, cytokines, and chemokines produced and released by skeletal myocytes such as interleukins (IL‐6, IL‐7, IL‐8, and IL‐15), brain‐derived neurotrophic factor (BDNF), angiopoietin‐like 4, myostatin, and irisin are thought to be involved in interactions between the muscle and the brain, which may lead to physiological and pathological processes of cognitive functions.^6^ For instance, myokine signaling may also explain the beneficial effects of physical activity on cognition in older adults, with an increase in the activity of the prefrontal cortex and hippocampus, which are two brain regions implicated in memory and cognition.^7^, ^8^, ^9^ However, these relationships between muscle and the brain were demonstrated only in healthy subjects. Therefore, more recent works have tried to explore associations between the brain and cognitive impairment in patients with sarcopenia. Several studies have examined the intricate connections between muscle functions and brain structures. A longitudinal investigation discovered that subtle abnormalities in total brain volume served as predictors for a decline in gait speed, though no significant correlation was found between handgrip strength and total brain volume.^10^ White matter changes have also been scrutinized, with a prior study revealing a connection between increased burdens of total and periventricular white matter hyperintensity (WMH) and a decline in gait performance over time.^11^ Upon examining the relationship with muscle mass, a recent large longitudinal study reported that the sarcopenia group exhibited significantly greater atrophy in the parietal area compared to the control group.^12^ In addition, a recent study revealed a significant correlation between retention of amyloid‐beta (Aβ) and muscle mass in elderly females without dementia.^13^ Although several previous studies have suggested distinctive relationships between brain structural/molecular changes and muscle mass/function in patients with sarcopenia, their results were occasionally inconsistent, and only partially elucidate the precise pathological process and its impact on cognitive impairment in sarcopenia. These results might be attributable to the lack of comprehensive predictive models involving whole‐brain structural changes and cerebral Aβ retention with decent sample sizes. Indeed, as cognitive impairments in older adults are influenced by intricate changes in brain pathology, the retention of cerebral Aβ, and alterations in gray and white matter may collectively act as dynamic neuropathological factors contributing to cognitive impairment in sarcopenia.

The aim of this study was to establish prediction models for cognitive impairment in sarcopenia integrating multiple neuroimaging methods such as structural MRI and amyloid positron emission tomography (PET) and sarcopenia sub‐domains like muscle mass and functions. Using these prediction models, we hypothesized that cognitive impairment of sarcopenia in non‐demented older adults might be explained by the level of cerebral amyloid retention, as well as gray and white matter changes as demonstrated on multimodal neuroimaging methods.

## 2 METHODS

### 2.1 Study participants

A total of 528 older adults without dementia was included in this study. Study participants were volunteers registered in the Catholic Aging Brain Imaging database (CABID), which contains MRI and PET scans and clinical data of the older adults who visited the outpatient clinic at the Catholic Brain Health Center, Yeouido St. Mary's Hospital, The Catholic University of Korea, from 2018 to 2023. The inclusion criteria of this study were subjects older than 60 years without dementia as verified by Clinical Dementia Rating (CDR) ≤ 0.5. Subjects with (1) another major psychiatric disorder, including schizophrenia, major depressive disorder, bipolar disorder, anxiety disorder, and substance use disorder; (2) a history of head trauma; and (3) any structural lesion in the central nervous system evidenced by MRI, such as malignancy, intracranial hemorrhage, infarction, or hydrocephalus.

### 2.2 Sarcopenia measures

In this study, we used three well‐established measures corresponding to the three core components of sarcopenia: skeletal muscle index (SMI) for assessing muscle mass, hand grip strength (HGS) for measuring muscle strength, and five times sit‐to‐stand test (5STS) for evaluating muscle function. SMI was derived from the results of bioimpedance analysis (BIA), HGS was determined using a spring hand dynamometer, and 5STS was conducted under the supervision of our research team (*Bioimpedance analysis, Hand grip strength test*, and *Five times sit‐to‐stand test* subsections in the Supplementary Method). According to the criteria outlined by the Asian Working Group for Sarcopenia 2019 (AWGS‐2019), an individual is diagnosed with sarcopenia if the individual exhibits low muscle mass (SMI < 7.0 kg/m^2^ for men, 5.7 kg/m^2^ for women), along with low muscle strength (HGS ≤ 28 kg for men and 18 kg for women), and/or low physical performance (5STS ≥ 12 s).^14^

**RESEARCH IN CONTEXT**

1. **Systematic review**: The authors reviewed the broad spectrum of published literature covering medical and non‐medical fields using traditional search engines such as PubMed and reference sections from related papers from the prior works to collect relevant articles to sarcopenia and cognition. There are few studies using multi‐modal neuroimaging and constructing comprehensive model dealing with the impact of sarcopenia on cognitive impairment in the non‐demented older adults.
2. **Interpretation**: Our findings not only replicate the existing relationship between sarcopenia and cognitive impairment in the elderly, also suggest that each sarcopenia measure reflecting muscle mass, power, and speed have distinctive effects on brain structure and amyloid‐beta (Aβ) retention. Cortical thickness was significantly associated with muscle power (hand‐grip strength, HGS; left superior temporal cortex) and speed (five‐time stand‐to‐sit test, 5STS; bilateral precentral and insula cortex), while Aβ was associated with muscle mass (skeletal muscle index, SMI). Furthermore, brain atrophy was primarily influenced by SMI and HGS through Aβ retention and periventricular white matter hyperintensity (WMH), respectively. The 5STS directly affected brain atrophy, contributing to cognitive decline.
3. **Future directions**: To prevent cognitive decline associated with muscle loss, it is essential to consider treatments targeting each component of sarcopenia. Subsequent longitudinal studies or randomized clinical trials for sarcopenia intervention should incorporate multimodal neuroimaging with cognitive measures to further confirm the causal connection between sarcopenia and brain pathologies.

### 2.3 Cognitive outcome and other clinical measures

The cognitive functions of the participants were evaluated using the Korean version of the Consortium to Establish a Registry for Alzheimer's Disease Assessment Packet (CERAD‐K) neuropsychological battery.^15^ This comprehensive test assesses several cognitive domains including memory, visuospatial construction, language, attention, and executive functions. The memory domain consists of word list memory (WLM), word list recall (WLR), word list recognition (WLRc), and constructional praxis recall (CPR), the visuospatial domain is assessed through constructional praxis (CP), the language domain includes 15‐item Boston Naming test, the executive function domain contains the verbal fluency (VF) test, and global cognition is measured by the Mini‐Mental State Examination (MMSE‐KC).^16^ The total score of CERAD‐K was calculated by adding all scores of subtests excluding the MMSE‐KC score.

In addition, we also collected data on depression and *apolipoprotein E* (*APOE)* genotypes, which may act as confounding factors in the cognitive function of older adults. The severity of depression was evaluated using the 17‐item Hamilton depression rating scores (HAMD_17_). *APOE* genotyping was performed using DNA extracted from participants’ peripheral blood during their initial visit to our clinic. Detailed descriptions of the HAMD_17_ and *APOE* genotyping procedures are available in the Supplementary Materials. (See the *Hamilton Depression Rating Scale* and *APOE genotyping* in the Supplementary Method section.)

### 2.4 Image acquisition and processing

Structural T1‐weighted and T2*‐weighted fluid‐attenuated inversion recovery (FLAIR) MRI images and amyloid PET images were obtained. All subjects underwent MRI; among them, 265 individuals also underwent amyloid PET imaging. MRI images were scanned using a Siemens Skyra 3T scanner (Siemens Healthcare, Erlangen, Germany) equipped with a 20‐channel head and neck coil. Structural T1‐weighted images were obtained using a magnetization‐prepared rapid gradient echo scan (MPRAGE) sequence with the parameters as follows: repetition time (TR) = 1860 ms, echo time (TE) = 25.3 ms, flip angle = 9°, field of view (FOV) = 224 × 224 mm, matrix size of 256 × 256, 208 axial slices with a slice thickness of 1.0 mm. T2*‐weighted fluid attenuation inversion recovery (FLAIR) sequence with 0.6 × 0.6 × 5.0 mm^3^ voxel size were as follows: TR = 9000 ms, TE = 76.0 ms, flip angle = 150°, and FOV = 220 × 220 mm. Amyloid PET, Biograph 64, Vision 600 (Siemens Medical Solutions Inc., USA), was used to acquire the flutemetamol PET scans. Static PET scans were performed from 90 to 110 min after 185 MBq of flutemetamol injection, with the matrix size of 256 × 256, and the voxel size of 1.3364 × 1.3364 × 3 mm^3^. Acquired DICOM files were anonymized and converted to NIfTI format with “dcm2niix”.^17^

T1 structural MRI images were reconstructed and the values of cortical thickness were calculated by using the FreeSurfer image analysis suite (version 6.0, http://surfer.nmr.mgh.harvard.edu) as described in previously published literature.^18^, ^19^ Degree of white matter degeneration, especially deep and periventricular white matter hyperintensity (dWMH and pvWMH, respectively), relative to total brain volume was estimated from T2 FLAIR images using the AQUA 2.0 program (Neurophet, South Korea).^20^ The global amyloid standardized uptake value ratio (SUVR) was quantified by automated segmentation software based on deep‐learning model (SCALE PET v. 0.1.3.1 by Neurophet, South Korea)^21^ using pons as the reference region.^22^ A cutoff value of 0.62 for global SUVR was used to determine amyloid‐positivity, as described in previous studies.^23^, ^24^

### 2.5 Statistical analysis

Propensity score matching (PSM) was performed to eliminate the possible bias by confounding variables (age, education, sex, *APOE4* carrier status, and HAMD_17_ score) with “psmpy” package (version 0.3.13). When comparing demographic characteristics between groups containing individuals with or without sarcopenia, analysis of covariance (ANCOVA) was conducted with the same set of covariates. The chi‐squared test was applied to test the difference in categorical variables for demographic and clinical characteristics. The presence of a correlation was assessed using Spearman's rank correlation test, and a partial correlation test was performed, controlling age, sex, and education. All statistical analyses were performed with the “Scipy” package (version 1.10.0) and “Pingouin” package (version 0.5.3) using Python 3.10.9.

The general linear model (GLM) was implemented at each vertex in the whole brain to identify the brain regions in which the sarcopenia group showed significant differences in cortical thickness relative to the non‐sarcopenia group, using the FreeSurfer's “mri_glmfit” (described at http://surfer.nmr.mgh.harvard.edu/fswiki/mri_glmfit). The correlation analyses between regional cortical thickness/Aβ retentions and sarcopenia subcomponents were also conducted. In particular, vertex‐wise GLM analyses were performed to explore the brain regions that showed significant correlations between cortical thickness/Aβ retentions and sarcopenia subcomponents such as the SMI, the HGS, the 5STS across all subjects, also using “mri_glmfit” function. We controlled for the effects of age, education, and sex in all general linear model analyses performed. For multiple comparisons correction, family‐wise error correction *p *< 0.05 using the Monte Carlo Null‐Z simulation with 10,000 permutations was applied.

The impacts of observed and hidden variables related to imaging biomarkers, including global Aβ burden, regional atrophy, and white matter lesion burden were examined with a path model. We used partial least squares structural equation modeling (PLS‐SEM) to analyze the direct and indirect effects of these variables on sarcopenia and cognitive outcome measures.^25^ Four latent variables—“cognition,” reflected by CERAD‐K scores; “atrophy,” consisting of the mean values of cortical thickness from both hemispheres and averaged bilateral hippocampal volume; “Aβ retention,” which was linked to global mean standardized value uptake ratio (SUVR); and “pvWMH” represented by the proportion of the volume of pvWMH—were introduced to the inner model. Our analysis focused on testing predefined biomarker pathways as illustrated in Figure S1. Detailed methods and results for the path model are described in the Supplementary Materials.

## 3 RESULTS

### 3.1 Demographics and clinical characteristics

The demographic and clinical characteristics of study participants are summarized in Table 1. Among 528 total subjects, 130 (60 males and 70 females) met the criteria for sarcopenia (24.6%). The mean age was significantly older in the sarcopenia group, but there was no difference in education, sex, or *APOE4* carrier status between the non‐sarcopenia and sarcopenia groups. The level of two diagnostic components of sarcopenia, such as SMI and HGS, were significantly lower, and the log‐transformed 5STS scores were higher in the sarcopenia group. The CERAD‐K scores were also significantly lower, and a greater number of individuals with MCI was included in the sarcopenia group. Regarding imaging biomarkers, the sarcopenia group showed significantly thinner mean cortical thickness compared to the non‐sarcopenia group. However, there were no significant differences in the deep, periventricular, and total WMH volumes or the amyloid PET global mean SUVR between the two groups.

**TABLE 1:** Demographic and clinical characteristics of study participants.

Parameter	All (*n* = 528)	Without sarcopenia (*n* = 398)	With sarcopenia (*n* = 130)	Statistics (*p*‐value)
Age (years)	76.24 ± 7.07	74.66 ± 6.71	81.09 ± 5.84	*F *= 90.99 (*p *< 0.001*)
Education (years)	12.09 ± 4.52	12.28 ± 4.33	11.51 ± 5.05	*F *= 0.54 (*p *= 0.463)
Sex (M:F)	264:264	204:194	60:70	$\chi^{2}$=0.83 (*p *= 0.363)
*APOE* allele				$\chi^{2}$=0.59 (*p *= 0.442)
ε4 carrier	150 (28.4%)	117 (29.4%)	33 (25.4%)	
ε4 non‐carrier	378 (71.6%)	281 (70.6%)	97 (74.6%)	
HAMD_17_ total score	7.11 ± 5.24	6.96 ± 5.29	7.55 ± 5.09	*F *= 1.14 (*p *= 0.287)
Sarcopenia components				
Skeletal muscle index (kg/m^2^)	150 (28.4%)	117 (29.4%)	33 (25.4%)	*F *= 263.36 (*p *< 0.001*)
Hand grip strength (kg)	378 (71.6%)	281 (70.6%)	97 (74.6%)	*F *= 135.55 (*p *< 0.001*)
Five times sit‐to‐stand test (s)^a^	150 (28.4%)	117 (29.4%)	33 (25.4%)	*F *= 17.90 (*p *< 0.001*)
Cognitive status				$\chi^{2}$=32.23 (*p *< 0.001*)
CU	242 (45.8%)	214 (53.8%)	28 (21.5%)	
MCI	286 (54.2%)	184 (46.2%)	102 (78.5%)	
CERAD‐K total score	60.22 ± 14.86	62.69 ± 14.62	52.66 ± 12.96	*F *= 8.02 (*p *= 0.005*)
VF	12.56 ± 4.95	13.20 ± 4.95	10.58 ± 4.38	*F* = 3.70 (*p *= 0.055)
BNT	11.13 ± 2.76	11.55 ± 2.56	9.86 ± 2.99	*F* = 8.69 (*p *= 0.003*)
MMSE‐KC	25.24 ± 3.61	25.86 ± 3.35	23.35 ± 3.72	*F* = 15.99 (*p *= 0.000*)
CP	9.84 ± 1.41	9.94 ± 1.34	9.55 ± 1.58	*F* = 0.14 (*p *= 0.705)
WLM	15.28 ± 4.49	15.97 ± 4.41	13.16 ± 4.07	*F* = 4.13 (*p *= 0.043*)
WLR	4.25 ± 2.39	4.62 ± 2.33	3.12 ± 2.20	*F* = 5.52 (*p *= 0.019*)
WLRc	7.45 ± 2.43	7.79 ± 2.23	6.42 ± 2.71	*F* = 7.70 (*p *= 0.006*)
CPR	5.04 ± 3.41	5.51 ± 3.43	3.58 ± 2.92	*F* = 3.93 (*p *= 0.048*)
Mean cortical thickness (mm)	2.28 ± 0.16	2.31 ± 0.14	2.20 ± 0.17	*F *= 9.37 (*p *= 0.002*)
Total WMH (%)	0.91 ± 0.96	0.81 ± 0.97	1.18 ± 0.90	*F *= 0.67 (*p *= 0.414)
DWMH (%)	0.16 ± 0.24	0.15 ± 0.23	0.19 ± 0.24	*F *= 0.01 (*p *= 0.915)
pvWMH (%)	1.65 ± 1.78	1.47 ± 1.78	2.18 ± 1.66	*F *= 0.82 (*p *= 0.367)
Amyloid PET global SUVR (*n* = 265)	0.52 ± 0.15	0.52 ± 0.14	0.55 ± 0.17	*F *= 0.36 (*p *= 0.547)

*Note*: The data are presented as **“**mean ± SD” format for continuous variables and “counts (proportion in percentage)” format for categorical variables.

Abbreviations: BNT, 15‐item Boston Naming Test; CERAD‐K, Korean version of the Consortium to Establish a Registry for Alzheimer's Disease Assessment Packet; CP, constructional praxis; CPR, constructional praxis recall; CU, cognitively unimpaired; DWMH, deep white matter hyperintensity; HAMD_17_, 17‐item Hamilton Depression Rating Scale; MCI, mild cognitive impairment; MMSE‐KC, Korean version of mini mental status examination in the Korean version of CERAD Assessment Packet; PET, positron emission topography; pvWMH, periventricular white matter hyperintensity; SUVR, standardized uptake value ratio.; VF, verbal fluency; WLM, word list memory; WLR, word list recall; WLRc, word list recognition; WMH, white matter hyperintensity.

^a^ log‐transformed value was used due to the normality of distribution.

### 3.2 Correlational analysis

**FIGURE 1:**
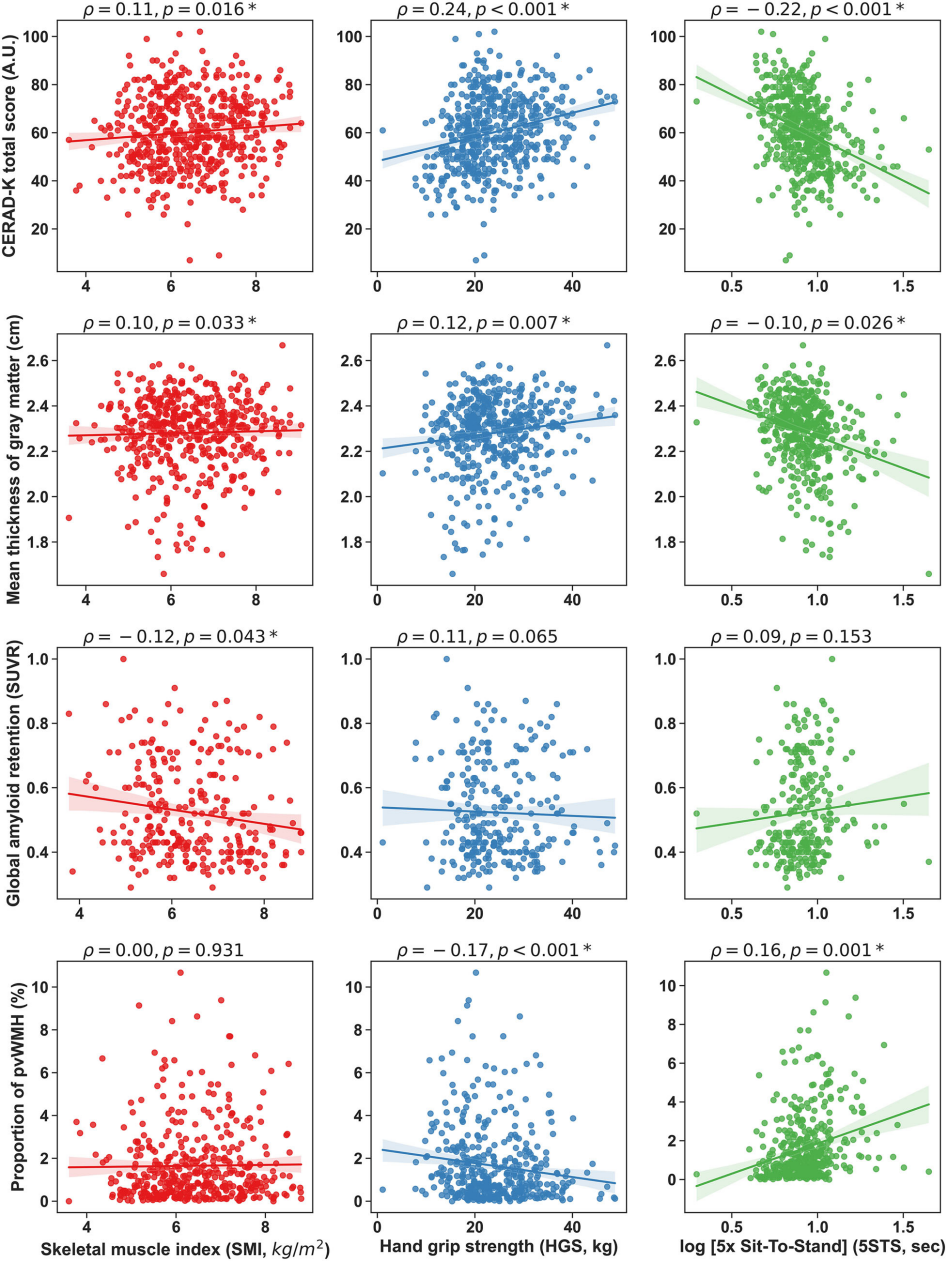
Correlational analysis results showing the relationship between the sarcopenia subcomponents and cognitive function, amyloid beta retention, and white matter change. All values of Spearman's correlation coefficient ρ are partial correlations controlling age, gender, and educational years. CERAD‐K, Korean version of the Consortium to Establish a Registry for Alzheimer's Disease Assessment Packet; pvWMH, periventricular white matter hyperintensity; SUVR, standardized uptake value ratio.

Figure 1 shows significant associations between all three sarcopenia subcomponents and cognitive scores across all subjects. There were significant positive partial correlations between the CERAD‐K total score and SMI (Spearman's *ρ* = 0.11, *p *= 0.016) and HGS (*ρ* = 0.24, *p *< 0.001). In addition, the 5STS results showed a negative partial correlation with the total CERAD‐K score (ρ= −0.22, *p *< 0.001). The mean cortical thickness was significantly correlated with SMI (ρ= 0.10, *p *= 0.033), HGS (ρ= 0.12, *p = *0.007), and 5STS (ρ= −0.10, *p *= 0.026).

The global mean amyloid SUVR showed a significant negative partial correlation with SMI (ρ= −0.12, *p *= 0.043), but not with HGS (*p *= 0.065), or the 5STS result (*p *= 0.153). The pvWMH volume showed a significant negative correlation with HGS (*ρ *= −0.17, *p *< 0.001), but a positive correlation with the 5STS result (ρ= 0.16, *p *= 0.001). However, no significant correlation was observed between pvWMH volume and SMI. There were also no significant correlations between sarcopenia subcomponents and total, periventricular, and deep WMH volumes. Finally, there were no significant correlations between sarcopenia subcomponents and dWMH volume.

We also tested the correlations among three sarcopenia components for each gender (Figure S2). The strength of association between SMI and HGS was the highest, followed by correlation between HGS and 5STS and between SMI and 5STS (Figure S3). Partial correlations between sarcopenia measures and cognition and brain markers were compared in each subgroup for males and females. Both subgroups showed similar associative relationships, but the partial correlation between sarcopenia measures and imaging measures for MCT and pvWMH showed statistical significance only for males (Figure S4).

### 3.3 Voxel‐wise cortical thickness and amyloid retention analysis

**FIGURE 2:**
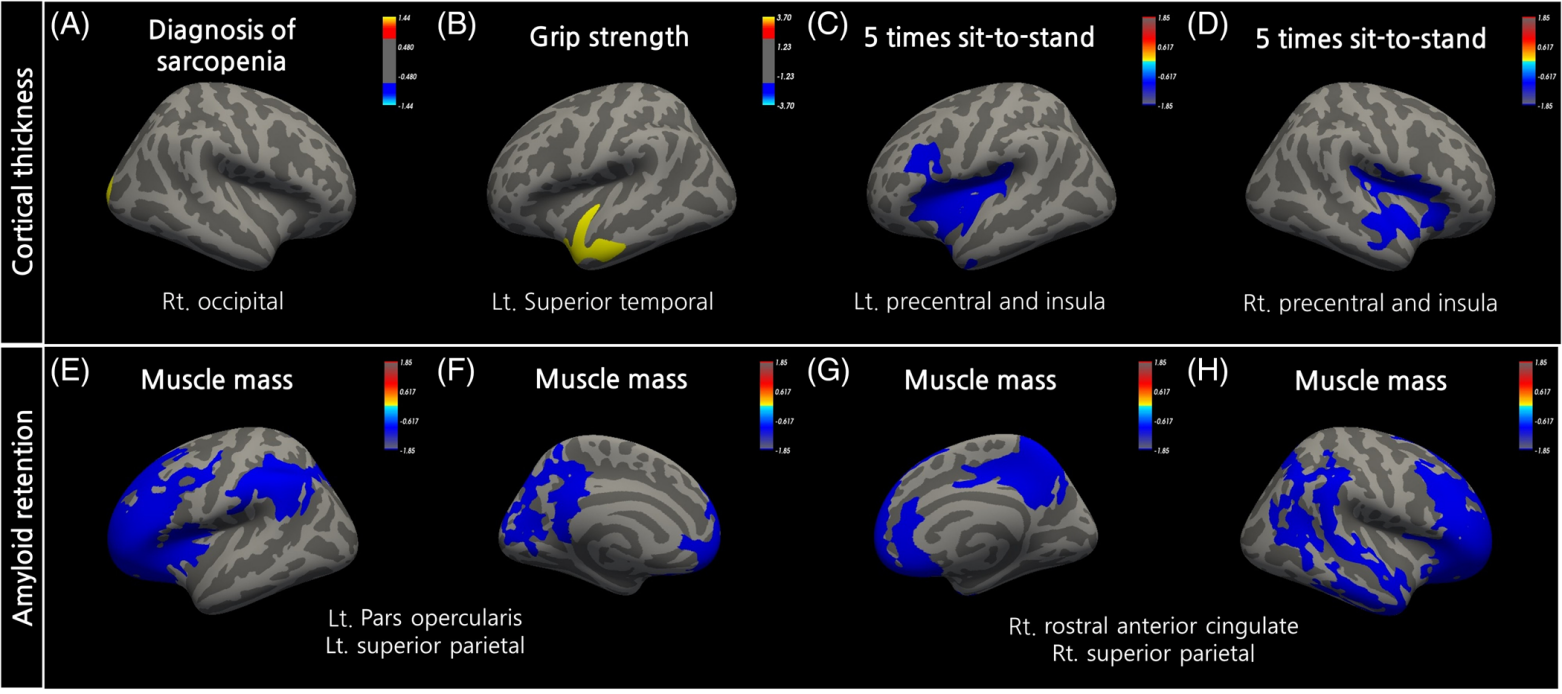
Statistical maps of surface‐based analysis for cortical thickness and amyloid retention. (A) Group difference between sarcopenia and non‐sarcopenia. Correlational analysis between cortical thickness and grip strength (B), 5 times sit‐to‐stand (C and D). (E–H) Correlational analysis between amyloid retention and muscle mass. Multiple comparison correction was conducted with Monte Carlo Z simulation with a threshold *p*‐value under 0.05. Only significant clusters are displayed with cluster‐wise *p*‐value < 0.05. Age, educational years, and intracranial volume (ICV) were controlled as covariates.

Group comparison for cortical thickness revealed that the right occipital pole was thinner in the sarcopenia group than in the non‐sarcopenia group after age, sex, and the intracranial volume (ICV) correction (*p *< 0.05, Figure 2A and Table 2). HGS showed a significant positive correlation with the left superior temporal cortical thickness (*p* < 0.05, Figure 2B). The cortical thickness of the bilateral precentral and insula areas showed a significant negative correlation with the 5STS (*p* < 0.05, Figure 2C,D, and Table 2). There was no significant correlation between regional cortical thicknesses and SMI. In the analysis of correlations between cerebral Aβ retention and the sarcopenia subcomponents, only SMI negatively correlated with Aβ retention in the bilateral frontal, superior parietal, anterior and posterior cingulate, and precuneus areas (Figure 2E–H and Table 2). All *p*‐values in this section were corrected by Monte Carlo z simulation.

**TABLE 2:** Results from surface‐based analysis for cortical thickness and amyloid retention.

Contrast	Hemisphere	Annotation	Max. t‐stat	Size (mm^2^)	MNI coordinates			CWP
Contrast	Hemisphere	Annotation	Max. t‐stat	Size (mm^2^)	X	Y	Z	CWP
Group differences in cortical thickness								
Non‐sarcopenia > sarcopenia	RH	Lateral occipital	3.494	1070.03	26.4	−94.7	5.5	0.0361
Correlational analysis between cortical thickness and sarcopenia subcomponents								
5STS	LH	Precentral	−4.417	5763.70	−39.9	−0.5	15.7	0.0002
5STS	LH	Precentral	−3.516	1093.21	−33.7	−15.6	43.8	0.0290
5STS	RH	Precentral	−6.844	4116.67	42.6	6.0	9.9	0.0002
HGS	LH	Superior temporal	3.875	1794.72	−50.9	4.0	−13.8	0.0002
Correlational analysis between amyloid beta retention and sarcopenia subcomponents								
SMI	LH	Pars Opercularis	−3.587	14872.42	−36.0	8.8	13.3	0.0002
SMI	LH	Superior parietal	−3.135	9978.50	−26.8	−62.8	37.8	0.0002
SMI	RH	Rostral anterior cingulate	−3.608	14591.49	5.5	30.4	−2.2	0.0002
SMI	RH	Superior temporal	−3.206	12947.98	56.1	−24.4	−1.6	0.0002

*Note*: Only significant clusters are displayed with cluster‐wise *p*‐value < 0.05. Multiple comparison correction was conducted with Monte Carlo Z simulation with vertices with *p*‐value under 0.05, and age, educational years, and intracranial volume (ICV) were controlled as covariates.

Abbreviations: 5STS, five times sit‐to‐stand test; CWP, cluster‐wise *p*‐value; HGS, hand‐grip strength test; LH, left hemisphere; MNI, Montreal Neurological Institute; RH, right hemisphere; SMI, skeletal muscle index.

### 3.4 Path modeling with partial least square structural equation modeling

**FIGURE 3:**
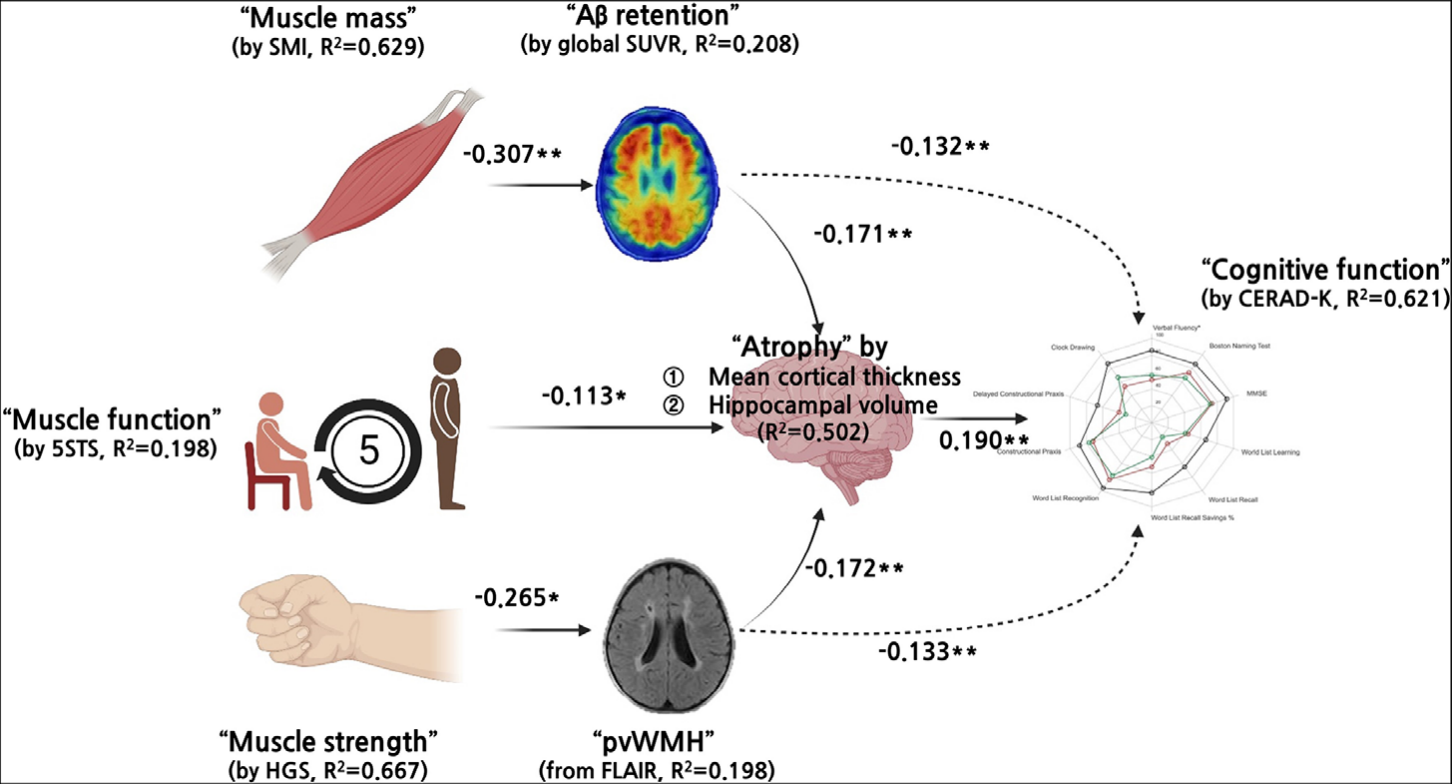
The path model explaining cognitive function and sarcopenia subcomponents with multiple brain pathology (brain atrophy, amyloid beta retention, and white matter change). The “atrophy” is an endogenous latent variable composed of cortical thickness for each hemisphere and hippocampal volume. Increased atrophy variable means thicker cortex and larger volume of the hippocampus. The “cognition” is also an endogenous latent variable reflecting the components of CERAD‐K. The “amyloid retention” is globally averaged value of SUVR, and “pvWMH” is representing the proportion of the volume of perivascular white matter hyperintensity divided by whole intracranial volume. * denotes *p *< 0.05, ** denotes *p *< 0.01, and *** denotes *p *< 0.001. 5STS, five time sit‐to‐stand test; CERAD‐K, Korean version of the Consortium to Establish a Registry for Alzheimer's Disease Assessment Packet; pvWMH, periventricular white matter hyperintensity; SUVR, standardized uptake value ratio.

We adapted partial least squares structural equation modeling to avoid multicollinearity between markers within the same domain and to test the direct and indirect effects of sarcopenia components (Figure 3 and Table S1). In the fitted model, the direct and negative effect of SMI on Aβ retention (β= −0.307, p= 0.007), that of HGS on pvWMH volumes (β= −0.265, p= 0.030), and that of the 5STS result on brain atrophy (β= −0.113, p= 0.042) were the only significant path coefficients between sarcopenia and neuroimaging measures. There were also significant direct effects of Aβ retention (β= −0.171, p= 0.002), WMH volumes (β= −0.172, p= 0.002), and brain atrophy (β= 0.190, p= 0.001) on cognition and indirect effects of Aβ and pvWMH volume on cognition mediated by brain atrophy (relationship of pvWMH to atrophy, β= −0.133, p= 0.006; relationship of Aβ to atrophy, β= −0.132, p= 0.006).

The explained variance (*R* ^2^) of the latent variable for cognition was higher than the moderate level at 0.621, and those of sarcopenia‐related measures such as HGS (*R* ^2 ^= 0.667) and SMI (*R* ^2 ^= 0.629) but except for the 5STS result (*R* ^2 ^= 0.198), were also higher. Among neuroimaging markers, the explained variance for brain atrophy was highest (*R* ^2 ^= 0.502), followed by that for Aβ retention (*R* ^2 ^= 0.208) and then pvWMH volume (*R* ^2 ^= 0.198). The goodness‐of‐fit value was large at 0.534, indicating the sufficient validity of the path model. More specific results like estimated effects, weights, and loadings, as well as model summaries, are given in our Supplementary Materials (Figure S5 and Tables S1–S3).

## 4 DISCUSSION

To the best of our knowledge, this is the first study to establish comprehensive prediction models for cognitive impairment in sarcopenia integrating multiple brain pathologies such as amyloid, vascular, and neurodegeneration, and sarcopenia sub‐domains with decent sample sizes. Although there has been concern about the potential for spurious correlations between sarcopenia and cognitive function due to the existence of multiple clinical confounders (e.g., age, education, sex, *APOE* genotype, and depression),^26^ we identified differential paths linking multiple brain pathologies, sarcopenia, and cognitive impairment in non‐demented older adults. The lower SMI and HGS were associated with brain atrophy potentially via associations with Aβ retention and WMH, respectively, which could lead to cognitive function impairments. On the other hand, lower 5STS results directly affected brain atrophy without mediation by Aβ and WMH, contributing to cognitive function impairments. PLS‐SEM approach, which is more robust, less sensitive to sample size, and also capable of addressing multicollinearity issue, enabled us to find these possible pathways.^25^

In our study, the proportion of individuals diagnosed with sarcopenia was 26% (130 out of 528 subjects), which was slightly higher than in previous studies.^27^ The prevalence of sarcopenia could vary depending on the type of diagnostic criteria used or characteristics of populations recruited. A recent meta‐analysis showed that the global prevalence of sarcopenia diagnosed by European Working Group on Sarcopenia in Older People (EWGSOP) ranged from 10% (EWGSOP2) to 27% (EWGSOP) in older adults aged ≥ 60 years.^28^ Another meta‐analysis reported that the rate of sarcopenia was 13.1% when using EWGSOP criteria in South Koreans aged ≥ 65 years.^29^ However, a study by Kim et al., which adapted a diagnostic criteria similar to ours (AWGS‐2019) showed that the rate of sarcopenia was as high as 22.6% in community‐dwelling South Korean older adults aged ≥ 70 years.^30^ In addition, a recent meta‐analysis showed that the prevalence of sarcopenia in outpatient clinic settings was higher (23.2%) than community‐dwelling older adults (17.3%).^27^

We showed a significant association between the reduction of skeletal muscle mass and the accumulation of Aβ in several cortical regions such as the bilateral parietal, precuneus, and cingulate areas, in line with findings of previous studies.^13^, ^31^ A few prior works observed that lower thigh muscle mass^13^ and body mass index^31^ were significantly associated with cerebral Aβ retention in non‐demented older adults. Another population‐based Mendelian randomization study, controlling for genetic effects, reported a lower risk of AD incidence with a higher body mass.^32^ The purported molecular mechanisms linking muscle mass reduction and Aβ retention include increased insulin resistance and mitochondrial dysfunction.^33^ Lower skeletal muscle mass is associated with insulin resistance,^34^ which is a possible risk factor for cognitive impairment.^35^ In addition, mitochondrial dysfunctions are common in individuals with low muscle mass.^33^ These may lead to the overproduction of reactive oxidative species and their related oxidative stress, which in turn increase the secretion and production of Aβ in the brain.^36^ Since most studies supporting these hypotheses were conducted at the preclinical or cellular level, generalization to the human population requires careful interpretation. Nonetheless, our findings indicated that only skeletal muscle mass, not muscle strength or function, would be an important factor contributing to Aβ retention in the continuum of AD. Further, longitudinal studies are needed to evaluate the causality of the relationship between muscle mass and Aβ retention and the interplay of the underlying mechanisms.

We also observed the mediation effect of pvWMH and brain atrophy linking HGS and cognitive impairment in non‐demented older adults. This result is in accordance with those of previous studies showing associations between WMH and HGS in middle‐aged healthy^37^ and depressive individuals,^38^ as well as an elderly population with subjective cognitive decline.^39^ A previous large longitudinal cohort study observed reduced HGS to be associated with cognitive impairment, increased WMH volume and vascular dementia diagnosis.^37^ In addition, a randomized clinical trial documented the effect of resistance exercise training for enhancing HGS on WMH reduction.^40^ Although the underpinning mechanisms linking HGS, pvWMH, and cognitive impairment remain unclear, vascular endothelial dysfunction may play a pivotal role.^37^, ^41^, ^42^, ^43^ Furthermore, we included WMH as an imaging marker for cerebral small vessel disease (CSVD), there exists a variety of imaging markers such as cerebral microbleeding, dilated perivascular space, and lacune infarction.^43^ Indeed, as several vascular changes may impact cognitive function either directly or indirectly,^44^ more comprehensive models encompassing various vascular imaging markers are needed for a thorough understanding of the association between muscle strength and vascular dysfunction.

Regarding the 5STS, which is a proxy measure of gait speed, we found a significant association between 5STS and cognitive functions mediated by brain atrophy. In addition, 5STS was negatively correlated with the cortical thickness of the precentral gyrus and insular. Several previous studies have observed significant associations between gait speed and brain volume. Specifically, brain regions related to motor function, including the precentral gyrus, have been reported to show focal gray matter atrophy associated with gait speed.^45^ In addition, a large prospective cohort study demonstrated a positive correlation between the volume reduction of the insula and walking speed.^46^ The insula serves as a hub connecting various brain networks responsible for emotion, cognition, and motor functions.^47^, ^48^ Atrophy of these regions has been reported to be associated with various neurodegenerative diseases and subsequent cognitive impairment.^49^ Meanwhile, a relationship between 5STS and pvWMH, found in previous studies,^50^, ^51^ was also observed in our results from the correlation analysis but not the PLS‐SEM model. This trend might imply a potential spurious correlation or reverse causality between 5STS and pvWMH, or the existence of other paths reaching 5STS via brain atrophy or HGS. We did not include these paths because PLS‐SEM does not allow any cyclic structure. Further studies to delineate the existence and direction of the link between gait speed and WMH should be considered.

As our path model showed distinctive associations between sarcopenia sub‐components and brain pathologies, interventions aimed at preventing or treating sarcopenia, such as resistance training,^40^, ^52^ ambulatory rehabilitation,^53^ nutritional support,^53^, ^54^, ^55^, ^56^ and mixed programs,^57^ may have potential benefits for dementia prevention. Although sarcopenia is a preventable and modifiable factor even after middle age,^58^, ^59^ there is no well‐established treatment guideline for sarcopenia in the context of cognitive decline in the elderly. Multi‐domain life‐style intervention trials such as FINGER encompassed exercise counseling,^60^ but exercise interventions to mitigate or reverse the loss of muscle mass, strength, and speed were not included. This trend might be due to a lack of evidence supporting sarcopenia as a modifiable risk factor for cognitive impairment and dementia.

The limitations of our study include the following. First, as our path model was based on cross‐sectional data capturing relationships at a single point in time, it has a limited ability to interpret causal relations. Further longitudinal studies are needed to elucidate the causal relationship of sarcopenia sub‐domains, neuroimaging markers, and cognitive functions. Second, there was a group difference in age between the non‐sarcopenic and sarcopenic groups, although we used propensity score matching to reduce the effect of such confounding. Further, longitudinal studies with decent sample sizes might help to control this confounder. Third, information about other possible confounding variables related to lifestyle,^61^ such as smoking and alcohol use, or medical comorbidities like hypertension or diabetes mellitus, were not collected. This could also be addressed by adding relevant data fields to a future study. Fourth, we assumed linear relationships between sarcopenia and neuroimaging variables as well as between neuroimaging and cognitive variables. However, recent studies^62^ and our data (Figure S6) showed that the level of cerebral Aβ retention can have a non‐liner effect on cognitive function. Therefore, more comprehensive statistical models could be needed to overcome this issue.

In this study, our path model recapitulated the previously proposed mediating effects of neuroimaging biomarkers on cognitive impairments associated with sarcopenia. Sarcopenia sub‐domains including muscle mass, power, and speed showed distinctive associations between cognitive impairment and Aβ burden, WMH, and brain atrophy. Therefore, treatments targeting each sarcopenia sub‐domains should be considered, as they can partially alleviate the process of brain pathology and prevent the cognitive decline associated with sarcopenia.

## AUTHOR CONTRIBUTIONS

Sunghwan Kim contributed to the conceptualization, methodology, data curation, writing (original draft), visualization, and statistical analysis. Dong Woo Kang and Yoo Hyun Um contributed to the methodology, data curation, and writing (review and editing). Chang Uk Lee contributed to the conceptualization and supervision. Sheng‐Min Wang and HyunKook Lim contributed to the conceptualization, methodology, writing (review and editing), supervision, project administration, and funding acquisition. All authors contributed to the article and approved the submitted version.

## CONFLICT OF INTEREST STATEMENT

The authors declare that the research was conducted in the absence of any commercial or financial relationships that could be construed as a potential conflict of interest. Author disclosures are available in the Supporting Information.

## CONSENT STATEMENT

The study was reviewed and approved by the Institutional Review Board of The Catholic University of Korea (SC23RISI0100). All human subjects provided informed consent.

## Supporting information

Supporting Information

Supporting Information

## Data Availability

The datasets generated or analyzed during the current study are not publicly available due to Patient Data Management Protocol of Yeouido St. Mary's Hospital but are available from the corresponding author on reasonable request.
