# Supplementary material for: Development of a prediction model for cognitive impairment of sarcopenia using multimodal neuroimaging in non‐demented older adults
Source: Alzheimers Dement. 2024 Jun 18;20(7):4868–78. doi: 10.1002/alz.14054 (PMC11247690; doi:10.1002/alz.14054)
Supplement: Supplementary file 1 — Supporting Information [file ALZ-20-4868-s001.docx]

**Supplementary Materials for “Development of** **prediction model for cognitive impairment of sarcopenia using multimodal neuroimaging in non-dementing older adults” by Kim et al.**

**Supplementary Methods**

1. Bioimpedance analysis
2. Hand grip strength test
3. Five-times-sit-to-stand test
4. Hamilton Depression Rating Scale
5. *APOE* genotyping
6. Partial least square structural equation modeling (PLS-SEM)

**Supplementary Tables and Figures**

Supplementary Table 1. Effects in the Partial least square structural equation modeling (PLS-SEM) for cognition using sarcopenia subcomponents and brain pathology measures.

Supplementary Table 2. Weights and loadings of outer model in the Partial least square structural equation modeling (PLS-SEM).

Supplementary Table 3. Summary of inner model in the Partial least square structural equation modeling (PLS-SEM).

Supplementary Figure 1. A priori path model for the Partial least square structural equation modeling (PLS-SEM)

Supplementary Figure 2. Distributions of sarcopenia components.

Supplementary Figure 3. Correlational analysis results for three sarcopenia components

Supplementary Figure 4. Correlational analysis results for each gender showing the relationship between the subcomponents of sarcopenia and measure for brain pathology (cognition, amyloid beta retention, and white matter change)

Supplementary Figure 5. Path coefficients in the Partial least square structural equation modeling (PLS-SEM)

Supplementary Figure 6. The distribution of global SUVR of amyloid PET (A, B) and its correlation with cognitive function (C) in amyloid-positive (Aβ(+)) and amyloid-negative (Aβ(-)) subgroups.

**Supplementary References**

**Supplementary Methods**

*Bioimpedance analysis*

The Inbody 770 is employed to assess the skeletal muscle mass (SMM) in individuals. Following a 5-minute period of standing, adhering to the manufacturer's guidelines, participants stood barefoot on the scale of the device, aligning the soles of their feet with four corresponding electrodes. They gripped the handles with both hands, keeping contact with the electrodes on the thumbs and palms. Height, gender, and age information was inputted into the analysis software, and the device recorded the weight. Subjects remained stationary throughout the assessment. The skeletal muscle index (SMI) was calculated by dividing participant’s height from SMM.

*Hand grip strength test*

Participants’ hand grip strength (HGS) was measured by a trained research assistant using a Smedley spring hand dynamometer following standardized procedures. Briefly, participants were instructed to firstly select the most comfortable of five possible grip positions for the dynamometer. And then, participants were than advised to stand, keeping their elbow adjacent to their torso, and stretch the forearm to the floor with the thumb was facing forward. A maximal score was obtained from each person’s left and right hands.

*Five times sit-to-stand test*

Participants are instructed to sit on a chair at knee height with arms crossed over the chest and repeatedly stand up and sit back down as quickly as possible for a total of five times (“five times sit-to-stand”, or 5STS). The time taken is measured from the initiation of the first sit-down to the completion of the fifth standing up. Based on some data from a study for Asian [1] indicating that the time of 11.6 seconds for standing up from the chair five times is equivalent to a walking speed of 1.0 m/sec, so cut-off value is regarded as 12 seconds [2].

*Hamilton Depression Rating Scale*

The Hamilton Depression rating scale has been widely used to quantify the severity of depression in various clinical trials and studies for decades, after developed by Hamilton [5, 6]. This scale consists of 17 items for depressive mood, sleep difficulty, work and activity, psychomotor retardation and agitation, somatic and psychic anxiety, somatic symptoms, sexual symptoms, hypochondriasis, weight changes, and insights into disease evaluated by clinician. Its Korean-validated version was employed in our study [7].

*APOE genotyping*

To ensure the genotype of *APOE*, all participants’ DNA was isolated from the blood using the QIAmp Blood DNA Maxi Kit (Qiagen, Valencia, CA, USA). Genotypes for two *APOE* SNPs, rs429358 (E*4) and rs7412 (E*2), were determined using TaqMan SNP genotyping assays (Applied Biosystems, Foster City, California USA,). If a participant had at least one *APOE* ε4 allele, they were categorized as *APOE* ε4 carriers; if they had no *APOE* ε4 allele, they were categorized as *APOE* ε4 non-carriers.

*Partial least square structural equation modeling (PLS-SEM)*

PLS-SEM was estimated using “plspm” package (version 0.5.6) implemented with Python [8]. The statistical significance of each path coefficient was determined using the bootstrapping procedure with 5000 repetitions. Goodness-of-Fit (GoF) is used to evaluate the fitness of the model [8]. The GoF values are ranging between 0 and 1, and higher value indicate that the model better explains the empirical data [9], where values of 0.10 (small), 0.25 (medium), and 0.36 (large) indicate the global validity of the model [10]. Total explained variance of the PLS-SEM model was measured by the coefficient of determination (R^2^).

**Supplementary Tables**

**Supplementary Table 1. Effects in the Partial least square structural equation modeling (PLS-SEM) for cognition using sarcopenia subcomponents and brain pathology measures.**

| **Path** | **Estimate** | **Std. error** | **t** | **p>\|t\|** |
| --- | --- | --- | --- | --- |
| Sex 🡪 5STS | 0.178 | 0.061 | 2.895 | 0.004* |
| APOE4 🡪 5STS | 0.015 | 0.061 | 0.243 | 0.809 |
| Edu 🡪 5STS | -0.163 | 0.061 | -2.682 | 0.008* |
| Age 🡪 5STS | 0.388 | 0.061 | 6.348 | <0.001* |
| Sex 🡪 HGS | -0.778 | 0.040 | -19.648 | <0.001* |
| APOE4 🡪 HGS | 0.029 | 0.039 | 0.734 | 0.464 |
| Edu 🡪 HGS | -0.072 | 0.039 | -1.845 | 0.066 |
| Age 🡪 HGS | -0.395 | 0.039 | -10.033 | <0.001* |
| Sex 🡪 Mass | -0.775 | 0.042 | -18.569 | <0.001* |
| APOE4 🡪 Mass | -0.107 | 0.041 | -2.595 | 0.010* |
| Edu 🡪 Mass | 0.016 | 0.041 | 0.392 | 0.695 |
| Age 🡪 Mass | -0.221 | 0.042 | -5.315 | <0.001* |
| Sex 🡪 pvWMH | -0.308 | 0.112 | -2.746 | 0.007* |
| APOE4 🡪 pvWMH | 0.094 | 0.063 | 1.494 | 0.137 |
| Edu 🡪 pvWMH | -0.083 | 0.063 | -1.312 | 0.191 |
| Age 🡪pvWMH | 0.254 | 0.077 | 3.313 | 0.001* |
| STS 🡪 pvWMH | 0.044 | 0.069 | 0.629 | 0.530 |
| HGS 🡪 pvWMH | -0.265 | 0.121 | -2.191 | 0.030* |
| Mass 🡪 pvWMH | -0.007 | 0.112 | -0.059 | 0.953 |
| Sex 🡪 Ab | -0.095 | 0.111 | -0.855 | 0.393 |
| APOE4 🡪 Ab | 0.370 | 0.062 | 5.945 | <0.001* |
| Edu 🡪 Ab | -0.061 | 0.063 | -0.969 | 0.334 |
| Age 🡪 Ab | 0.107 | 0.076 | 1.403 | 0.162 |
| 5STS 🡪 Ab | 0.008 | 0.069 | 0.118 | 0.906 |
| HGS 🡪 Ab | 0.193 | 0.120 | 1.601 | 0.111 |
| Mass 🡪 Ab | -0.307 | 0.112 | -2.746 | 0.007* |
| Sex 🡪 Atrophy | 0.553 | 0.090 | 6.123 | <0.001* |
| APOE4 🡪 Atrophy | 0.021 | 0.054 | 0.384 | 0.701 |
| Edu 🡪 Atrophy | -0.079 | 0.050 | -1.577 | 0.116 |
| Age 🡪 Atrophy | -0.176 | 0.062 | -2.830 | 0.005* |
| 5STS 🡪 Atrophy | -0.113 | 0.055 | -2.047 | 0.042* |
| HGS 🡪 Atrophy | 0.055 | 0.098 | 0.566 | 0.572 |
| Mass 🡪 Atrophy | -0.039 | 0.091 | -0.433 | 0.665 |
| pvWMH 🡪 Atrophy | -0.172 | 0.054 | -3.188 | 0.002* |
| Ab 🡪 Atrophy | -0.171 | 0.054 | -3.157 | 0.002* |
| Sex 🡪 Cog | -0.037 | 0.053 | -0.712 | 0.477 |
| APOE4 🡪 Cog | -0.143 | 0.046 | -3.084 | 0.002* |
| Edu 🡪 Cog | 0.408 | 0.043 | 9.489 | <0.001* |
| Age 🡪 Cog | -0.363 | 0.048 | -7.592 | <0.001* |
| pvWMH 🡪 Cog | -0.133 | 0.048 | -2.789 | 0.006* |
| Ab 🡪 Cog | -0.132 | 0.048 | -2.778 | 0.006* |
| Atrophy 🡪 Cog | 0.190 | 0.059 | 3.227 | 0.001* |
| Dep 🡪 Cog | -0.156 | 0.043 | -3.580 | <0.001* |

*Abbreviations.* 5STS, five times sit-to-stand test; APOE4, carrier of apolipoprotein E (*APOE*) ε4 allele; Edu, education; HGS, hand grip strength; pvWMH, periventricular white matter hyperintensity; Ab, amyloid beta retention; Cog, cognition; Dep, depression.

**Supplementary Table 2. Weights and loadings of outer model in the Partial least square structural equation modeling (PLS-SEM).**

|  | **weight** | **loading** | **communality** | **redundancy** |
| --- | --- | --- | --- | --- |
| age | 1.000 | 1.000 | 1.000 | 0.000 |
| apet_average | 1.000 | 1.000 | 1.000 | 0.208 |
| apoe4 | 1.000 | 1.000 | 1.000 | 0.000 |
| atrophy_Hippocampus_volume | 0.385 | 0.772 | 0.596 | 0.299 |
| atrophy_lh_MeanThickness_thickness | 0.376 | 0.938 | 0.880 | 0.442 |
| atrophy_rh_MeanThickness_thickness | 0.374 | 0.938 | 0.880 | 0.442 |
| cerad_CPR | 0.168 | 0.820 | 0.672 | 0.417 |
| cerad_executive | 0.047 | 0.674 | 0.454 | 0.282 |
| cerad_general | 0.071 | 0.696 | 0.485 | 0.301 |
| cerad_language | 0.625 | 0.931 | 0.866 | 0.538 |
| cerad_memory | 0.296 | 0.676 | 0.457 | 0.284 |
| cerad_visuospatial | 1.000 | 1.000 | 1.000 | 0.000 |
| education | 1.000 | 1.000 | 1.000 | 0.000 |
| gender | 1.000 | 1.000 | 1.000 | 0.000 |
| sarcopenia_5sts | 1.000 | 1.000 | 1.000 | 0.198 |
| sarcopenia_bia | 1.000 | 1.000 | 1.000 | 0.629 |
| sarcopenia_grip_str | 1.000 | 1.000 | 1.000 | 0.667 |
| wm_pvwmh_pct | 1.000 | 1.000 | 1.000 | 0.198 |

**Supplementary Table 3. Summary of inner model in the Partial least square structural equation modeling (PLS-SEM).**

|  | **Type** | **R^2^** | **Adjusted R^2^** | **Block communality** | **Mean redundancy** | **AVE** |
| --- | --- | --- | --- | --- | --- | --- |
| APOE4 | Exogenous | 0.000 | 0.000 | 1.000 | 0.000 | 1.000 |
| Ab | Endogenous | 0.208 | 0.183 | 1.000 | 0.208 | 1.000 |
| Age | Exogenous | 0.000 | 0.000 | 1.000 | 0.000 | 1.000 |
| Atrophy | Endogenous | 0.502 | 0.481 | 0.785 | 0.394 | 0.785 |
| Cog | Endogenous | 0.621 | 0.606 | 0.587 | 0.364 | - |
| Edu | Exogenous | 0.000 | 0.000 | 1.000 | 0.000 | 1.000 |
| HGS | Exogenous | 0.000 | 0.000 | 1.000 | 0.000 | 1.000 |
| Mass | Endogenous | 0.667 | 0.661 | 1.000 | 0.667 | 1.000 |
| 5STS | Endogenous | 0.629 | 0.622 | 1.000 | 0.629 | 1.000 |
| Sex | Endogenous | 0.198 | 0.184 | 1.000 | 0.198 | 1.000 |
| pvWMH | Exogenous | 0.000 | 0.000 | 1.000 | 0.000 | 1.000 |

*Abbreviations.* AVE, Average variance extracted; APOE4, carrier of apolipoprotein E (*APOE*) ε4 allele; Ab, amyloid beta retention; Cog, cognition; Edu, education; HGS, hand grip strength; 5STS, five times sit-to-stand test; pvWMH, periventricular white matter hyperintensity.

**Supplementary Figures**

**Supplementary Figure 1. *A priori* path model for the Partial least square structural equation modeling (PLS-SEM)**


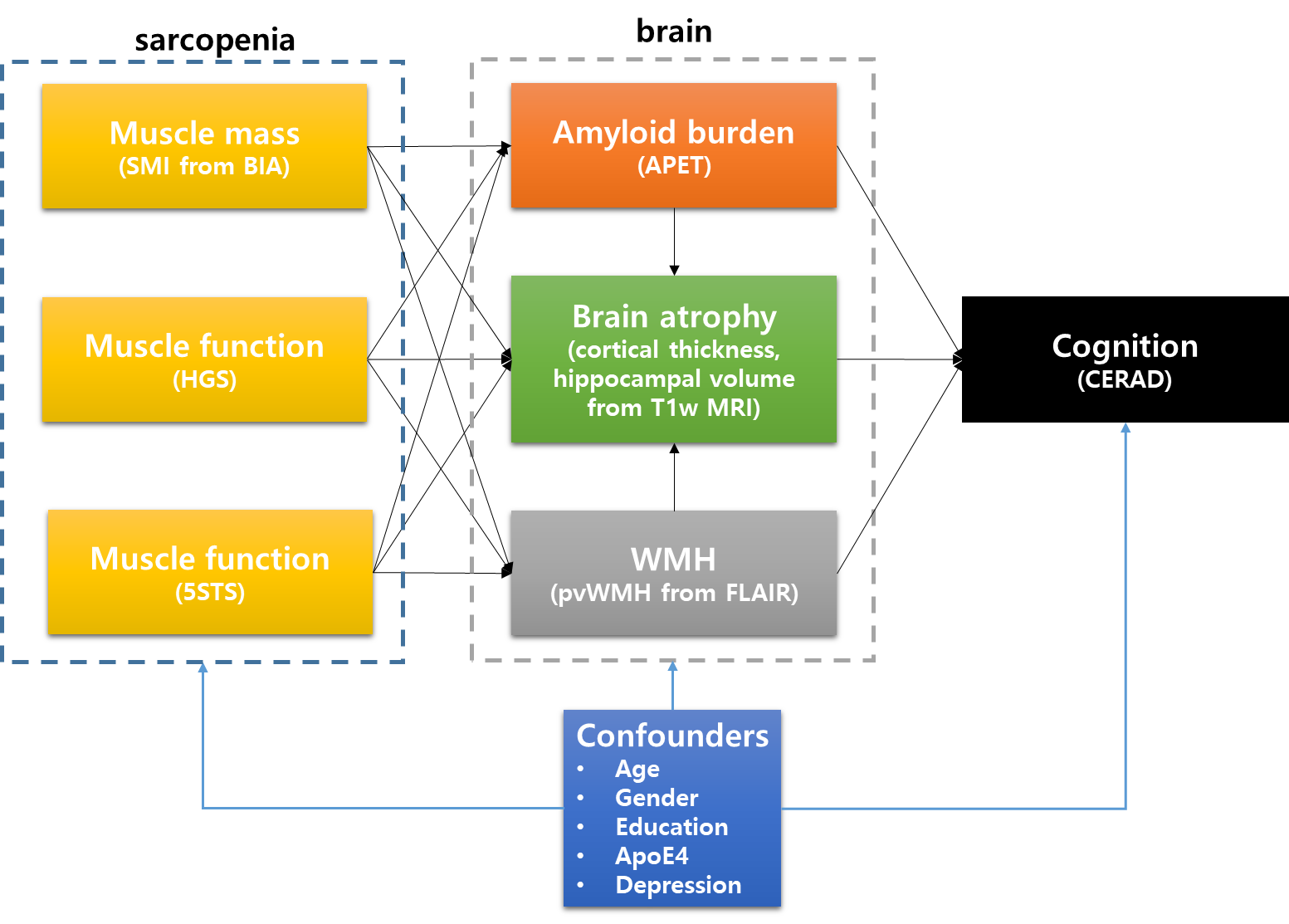


*Abbreviations*. SMI, skeletal muscle index; BIA, body impedance analysis; APET, amyloid positron emission topography; HGS, hand grip strength; T1w MRI, T1-weighted magnetic resonance imaging; CERAD, Korean version of the Consortium to Establish a Registry for Alzheimer's Disease Assessment Packet; 5STS, five-times-sit-to-stand test; WMH, white matter hyperintensity; PVWMH, periventricular white matter hyperintensity; FLAIR, Fluid attenuated inversion recovery.

**Supplementary Figure 2. Distributions of sarcopenia components.**


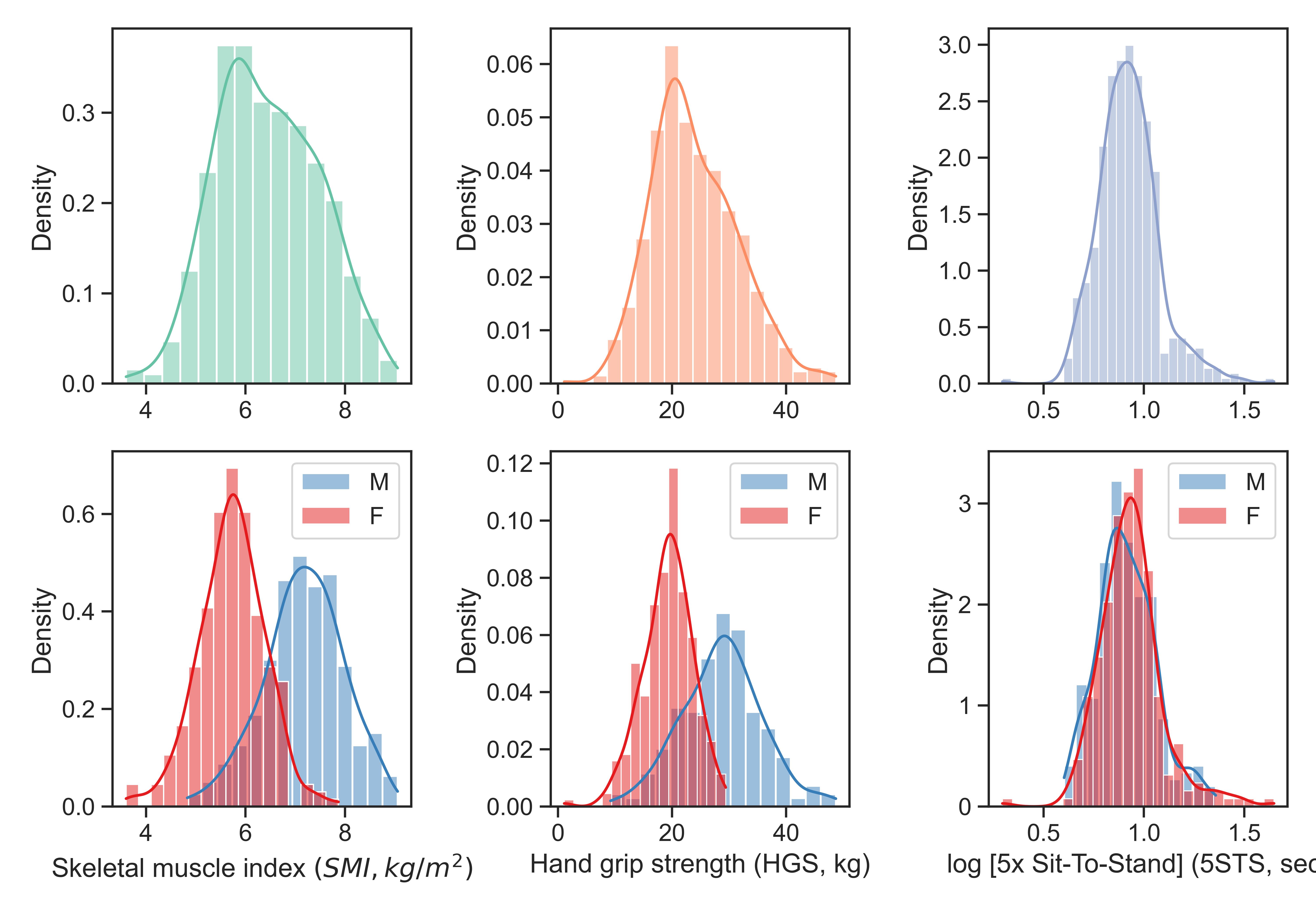


*Abbreviations*. M, male; F, female.

**Supplementary Figure 3. Correlational analysis results for three sarcopenia components.** All values of Spearman’s $\rho$ are partial correlation controlling age and educational years.


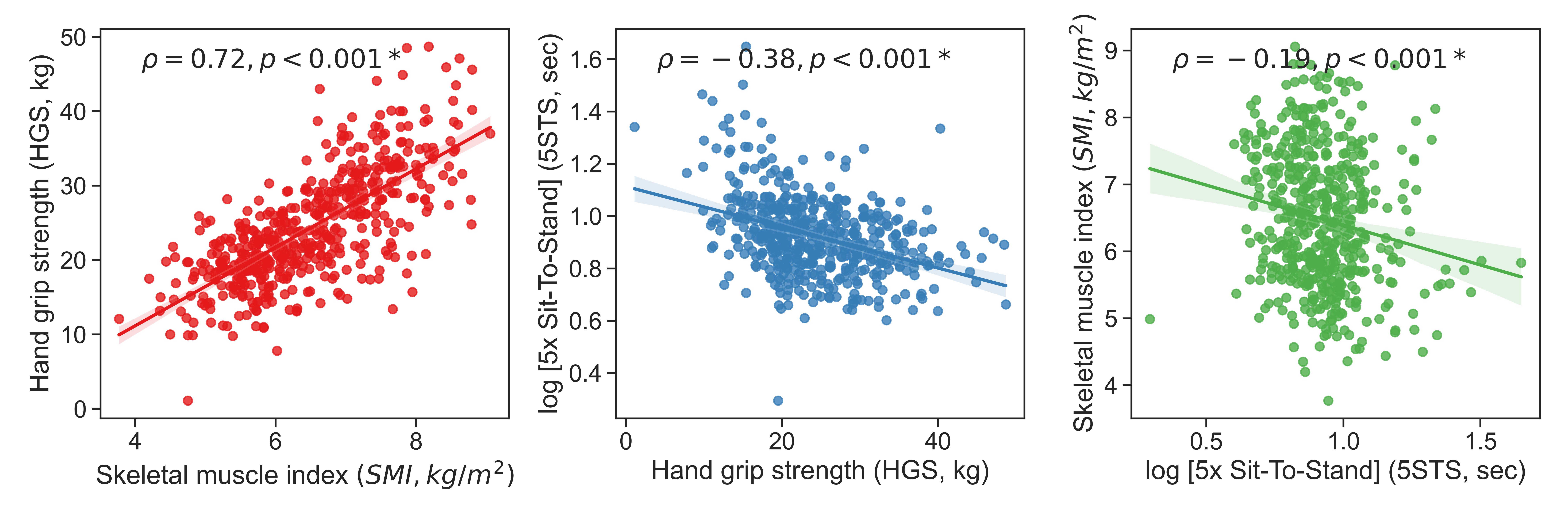


**Supplementary Figure 4. Correlational analysis results for each gender showing the relationship between the subcomponents of sarcopenia and measure for brain pathology (cognition, amyloid beta retention, and white matter change).** All values of Spearman’s $\rho$ are partial correlation controlling age and educational years.


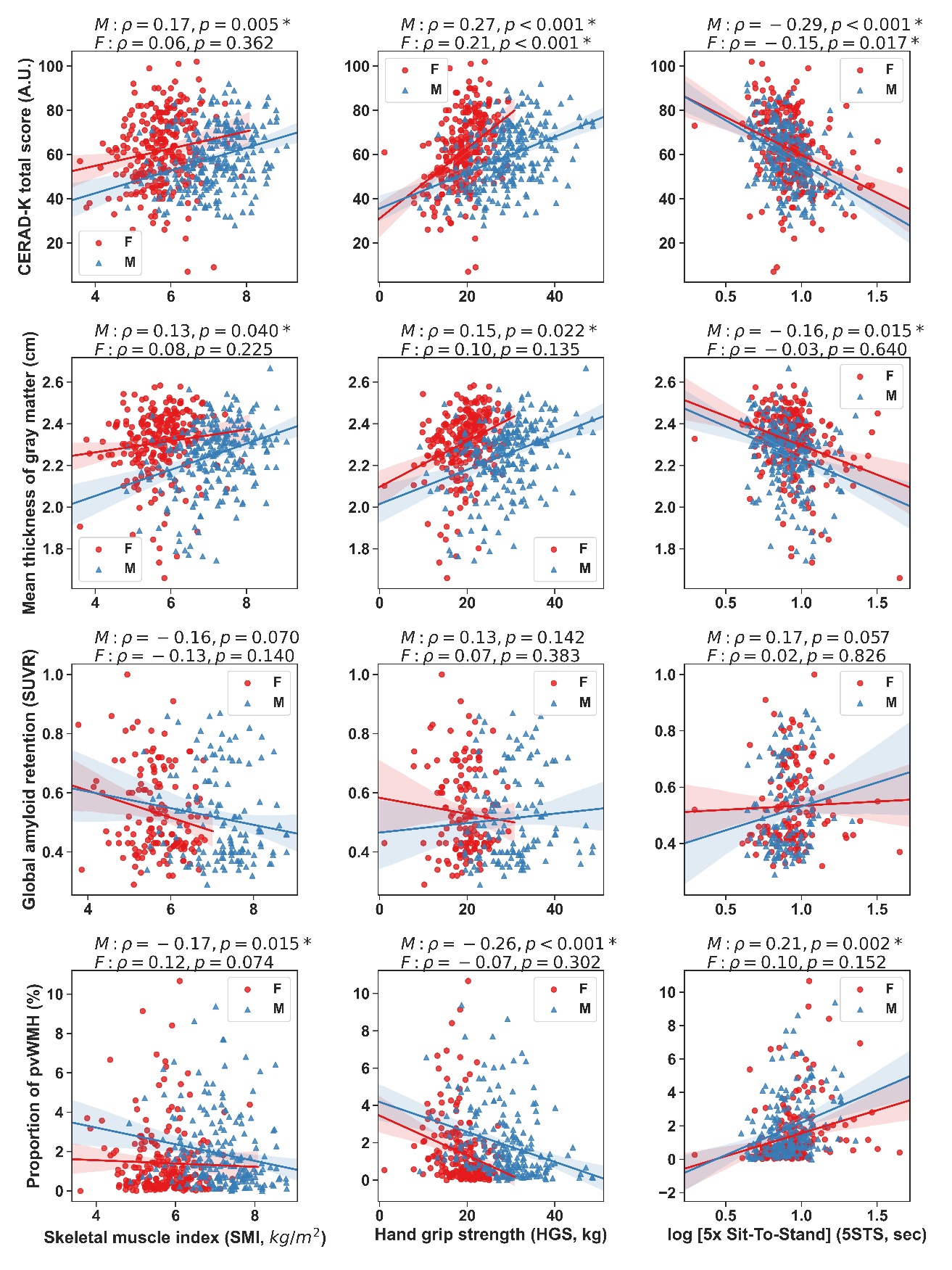


*Abbreviations*. M, male; F, female; CERAD-K, Korean version of the Consortium to Establish a Registry for Alzheimer's Disease Assessment Packet; A.U., arbitrary unit; SUVR, standardized uptake value ratio; pvWMH, periventricular white matter hyperintensity.

**Supplementary Figure 5. Path coefficients in the Partial least square structural equation modeling (PLS-SEM)**


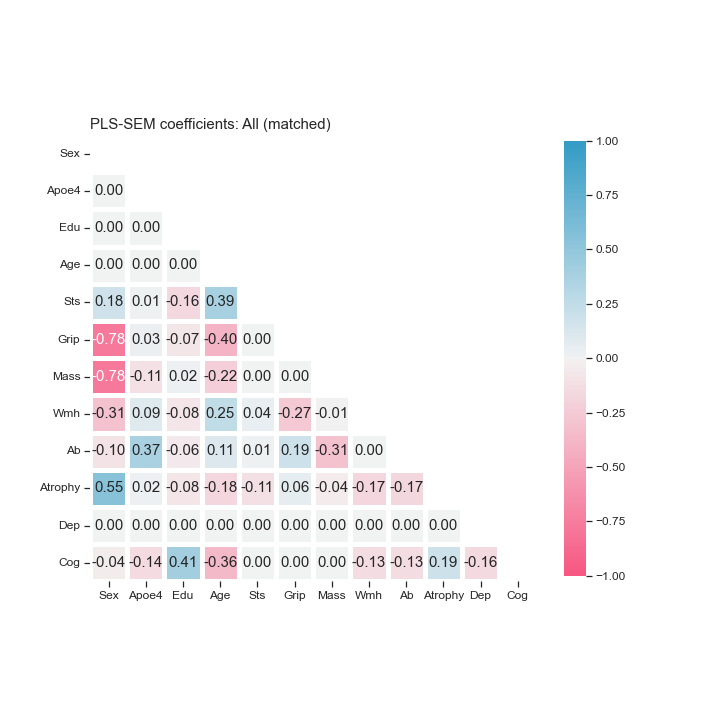


*Abbreviations.* Apoe4, carrier of apolipoprotein E (*APOE*) ε4 allele; Edu, education; Sts, five times sit-to-stand test; Wmh, periventricular white matter hyperintensity; Ab, amyloid beta retention; Dep, depression; Cog, cognition.

**Supplementary Figure 6. The distribution of global SUVR of amyloid PET (A, B) and its correlation with cognitive function (C) in amyloid-positive (Aβ(+)) and amyloid-negative (Aβ(-)) subgroups.** Global amyloid SUVR values displayed a left-skewed distribution (A) for both men and women (B). Depending on amyloid-positivity, there was different relationship between global standardized uptake value ratio (SUVR) and CERAD-K total score (C).


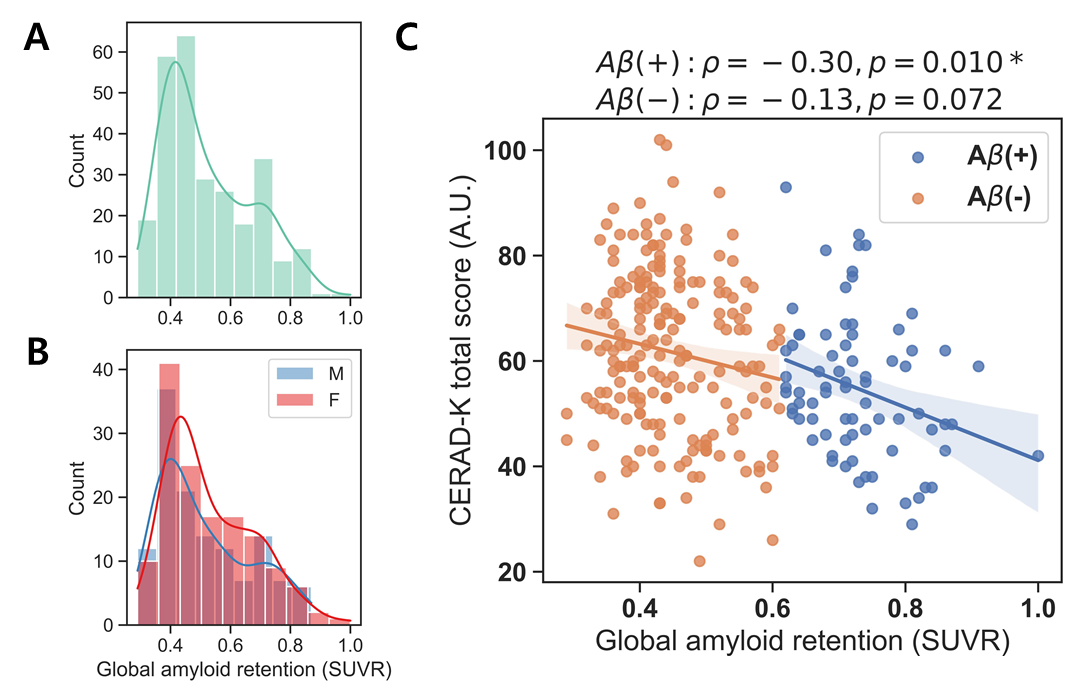


**Supplementary References**

[1] Nishimura T, Arima K, Okabe T, Mizukami S, Tomita Y, Kanagae M, et al. Usefulness of chair stand time as a surrogate of gait speed in diagnosing sarcopenia. Geriatrics & gerontology international. 2017;17:659-61.

[2] Chen L-K, Woo J, Assantachai P, Auyeung T-W, Chou M-Y, Iijima K, et al. Asian Working Group for Sarcopenia: 2019 consensus update on sarcopenia diagnosis and treatment. Journal of the American Medical Directors Association. 2020;21:300-7. e2.

[3] Lee JH, Lee KU, Lee DY, Kim KW, Jhoo JH, Kim JH, et al. Development of the Korean Version of the Consortium to Establish a Registry for Alzheimer's Disease Assessment Packet (CERAD-K) clinical and neuropsychological assessment batteries. The Journals of Gerontology Series B: Psychological Sciences and Social Sciences. 2002;57:P47-P53.

[4] JH P. Standardization of Korean version of the Mini-Mental State Examination (MMSE-K) for use in the elderly. Part II. Diagnostic validity. Korean J Neuropsych Assoc. 1989;28:125-35.

[5] Hamilton M. A rating scale for depression. Journal of neurology, neurosurgery, and psychiatry. 1960;23:56.

[6] Zimmerman M, Martinez JH, Young D, Chelminski I, Dalrymple K. Severity classification on the Hamilton depression rating scale. Journal of affective disorders. 2013;150:384-8.

[7] Yi J-S, Bae S-O, Ahn Y-M, Park D-B, Noh K-S, Shin H-K, et al. Validity and reliability of the Korean version of the Hamilton Depression Rating Scale (K-HDRS). Journal of Korean Neuropsychiatric Association. 2005:456-65.

[8] Hair JF, Risher JJ, Sarstedt M, Ringle CM. When to use and how to report the results of PLS-SEM. European business review. 2019;31:2-24.

[9] Tenenhaus M, Vinzi VE, Chatelin YM, Lauro C. PLS path modeling. Computational Statistics & Data Analysis. 2005;48:159-205.

[10] Wetzels M, Odekerken-Schroder G, van Oppen C. Using PLS path modeling for assessing hierarchical construct models: Guidelines and empirical illustration. Mis Quarterly. 2009;33:177-95.
